# Protein quality control and aggregation in the endoplasmic reticulum: From basic to bedside

**DOI:** 10.3389/fcell.2023.1156152

**Published:** 2023-04-19

**Authors:** Guofang Chen, Tingyi Wei, Furong Ju, Haisen Li

**Affiliations:** ^1^ Shanghai Key Laboratory of Maternal Fetal Medicine, Clinical and Translational Research Center of Shanghai First Maternity and Infant Hospital, Tongji University School of Medicine, Shanghai, China; ^2^ Ninth People’s Hospital, Shanghai Jiao Tong University School of Medicine, Shanghai, China; ^3^ Shanghai Institute of Precision Medicine, Shanghai, China; ^4^ Ming Wai Lau Centre for Reparative Medicine, Karolinska Institutet, Sha Tin, Hong kong SAR, China; ^5^ School of Life Sciences, Fudan University, Shanghai, China; ^6^ AoBio Medical, Shanghai, China

**Keywords:** ER, protein quality control, protein aggregate, phase transition, ER storage disease

## Abstract

Endoplasmic reticulum (ER) is the largest membrane-bound compartment in all cells and functions as a key regulator in protein biosynthesis, lipid metabolism, and calcium balance. Mammalian endoplasmic reticulum has evolved with an orchestrated protein quality control system to handle defective proteins and ensure endoplasmic reticulum homeostasis. Nevertheless, the accumulation and aggregation of misfolded proteins in the endoplasmic reticulum may occur during pathological conditions. The inability of endoplasmic reticulum quality control system to clear faulty proteins and aggregates from the endoplasmic reticulum results in the development of many human disorders. The efforts to comprehensively understand endoplasmic reticulum quality control network and protein aggregation will benefit the diagnostics and therapeutics of endoplasmic reticulum storage diseases. Herein, we overview recent advances in mammalian endoplasmic reticulum protein quality control system, describe protein phase transition model, and summarize the approaches to monitor protein aggregation. Moreover, we discuss the therapeutic applications of enhancing endoplasmic reticulum protein quality control pathways in endoplasmic reticulum storage diseases.

## Introduction

**FIGURE 1:**
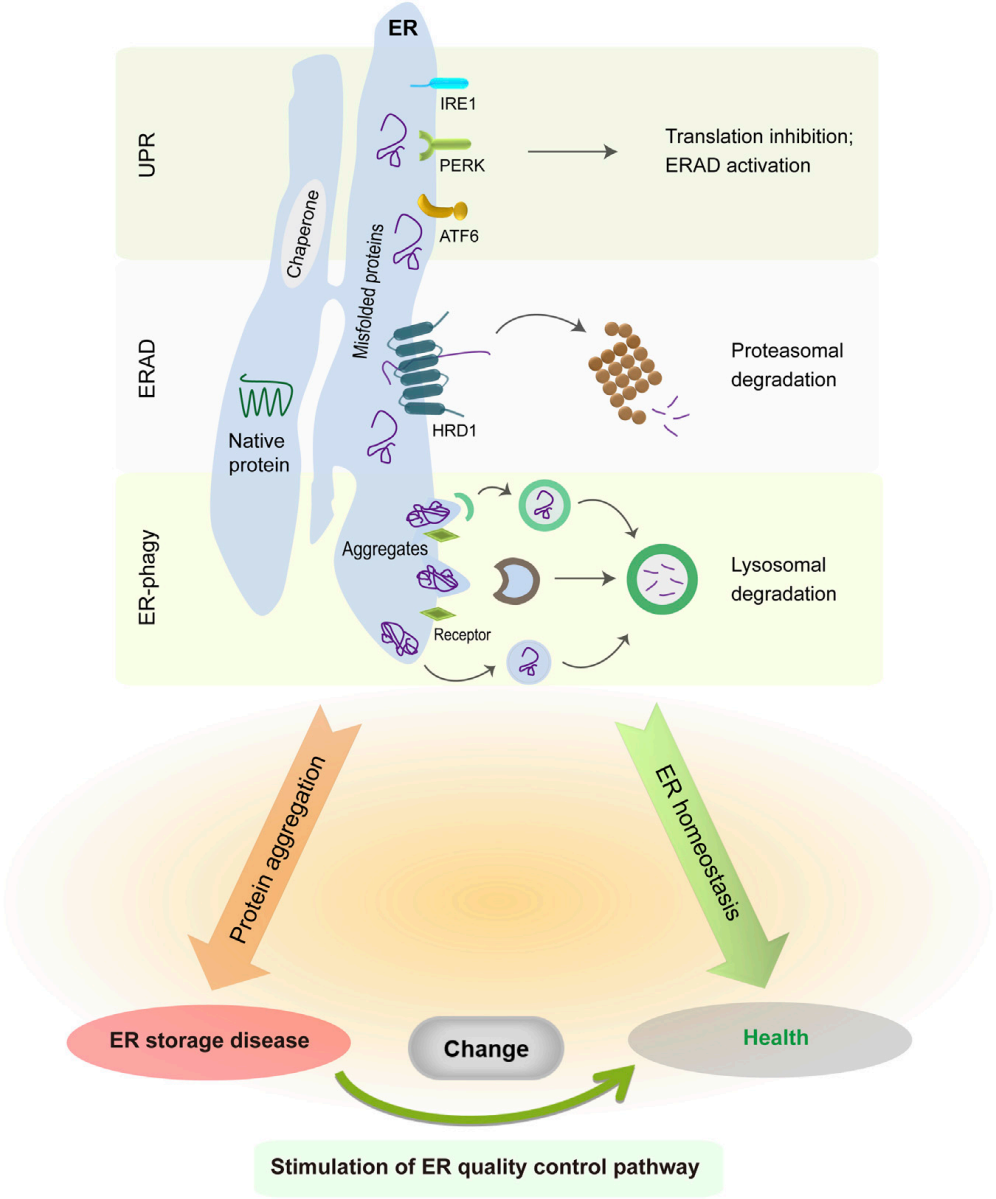
ER protein quality control system in normal and pathological conditions. The UPR is initiated by three distinct sensors, whose activation suppresses global protein translation but enhances ER folding and degradation capabilities. ER-resident faulty proteins can be transported to the cytosol via the ERAD complex and are degraded in the cytoplasmic proteasome. Meanwhile, insoluble protein aggregates are recruited to the ER-phagy receptors and then delivered into the lysosomes for degradation. The accumulation of misfolded protein aggregates in the ER causes illness, whereas enhancing ERAD or ER-phagy activity is sufficient to alleviate disease manifestations.

The ER is a single membrane-enclosed organelle within eukaryotic cells and plays crucial roles in diverse cell functions such as protein synthesis, lipid trafficking, calcium homeostasis, redox regulation, and cellular metabolism (Schwarz and Blower, 2016; Harada et al., 2021). Although the ER subunits have obvious differences in shape and functions, they are interconnected with each other to enable the free movement of luminal molecules throughout the ER. For instance, the calcium traveling freely inside the ER lumen may reach up to a high concentration from 60 to 400 μM (Bygrave and Benedetti, 1996; Rossi and Taylor, 2020), which facilitates proper folding and quality control of nascent proteins in the ER via the activation of calcium binding chaperones calreticulin (CRT) and calnexin (CNX). Due to its extremely large size, the ER accounts for a great proportion of the intracellular membrane system and has associations with nearly all other organelles (e.g., mitochondria and nucleus) by the membrane contact sites (Rowland and Voeltz, 2012; Matteis and Rega, 2015; Wu et al., 2018; Lee et al., 2020; Qin et al., 2020). The close connection between ER and mitochondria is critical in the maintenance of an oxidative environment inside the ER lumen (Csordás et al., 2006; Gilady et al., 2010; Booth et al., 2016; Yoboue et al., 2018), which benefits disulfide bond formation and oxidative protein folding. As such, the association between ER and other organelles has an important role in the modulation of cytosolic positioning and functions of cellular organelles. When facing extracellular and intracellular signals, the ER is able to undergo rapid changes in morphology and composition for the maintenance of ER homeostasis and cellular functions (Baumann and Walz, 2001; Schwarz and Blower, 2016). It has been estimated that ER-membrane-resident proteins and lipids are updated once every 10 days (Omura et al., 1967). Foremost, the ER is responsible for the biogenesis of appropriate one-quarter to one-third of all proteins in eukaryotic cells (Chen et al., 2013; Kumari and Brodsky, 2021). After nascent peptides move into the ER from cytosolic ribosomes, they are recognized by ER chaperones and folding enzymes to guide their proper assembly and posttranslational modifications. Once achieving native conformation, functional proteins leave the ER and travel to the Golgi via the secretion pathway. Since nascent proteins are vulnerable to errors, a proportion of them needs to be cleared owing to their intrinsically defective conformation. This process is implemented by ER protein quality control system comprising unfolded protein response (UPR), ER-associated protein degradation (ERAD), and ER-related autophagy (ER-phagy) (Shoulders et al., 2013; Schultz et al., 2018; Christianson and Carvalho, 2022; Wiseman et al., 2022; Chino and Mizushima, 2023; Thepsuwan et al., 2023). Although these three distinct pathways primarily work in parallel, all of them cooperatively function to remove ER-resident faulty proteins, decrease ER stress, and sustain ER proteostasis (Figure 1).

When ER protein quality control system is insufficient to clear misfolded proteins during pathological conditions, faulty proteins accumulate progressively and may form large protein aggregates, thereby disrupting ER homeostasis and cellular functions. Both ER stress and UPR dysfunction have been shown to play important roles in the etiology of many cancers and neurodegenerative disorders such as Alzheimer disease and Huntington disease (Imai et al., 2000; Saxena et al., 2009; Lee et al., 2010; Urra et al., 2016; Ajoolabady et al., 2022). Notably, ER stress responses are found to exert protective effects in the early disease progression with the aim to remove faulty proteins, but later exacerbate the neurodegeneration at the late stage (Hoozemans et al., 2009; Leitman et al., 2014; Mercado et al., 2016; Marcora et al., 2017). In fact, the accumulation and aggregation of faulty proteins happen within the cytoplasm of neurons in most cases of neurodegenerative diseases (Colla, 2019; Shacham et al., 2019; Bachmann et al., 2021), but leads to abnormal ER stress via the sequestration of ERAD factors, the impairment of ER-to-Golgi vesicle trafficking, the perturbation of UPR signaling cascades, and/or the disruption of ER calcium homeostasis (Yang et al., 2010; Abisambra et al., 2013; Hyrskyluoto et al., 2014; Hetz and Saxena, 2017; Remondelli and Renna, 2017; Betzer et al., 2018). Aberrant protein accumulation and aggregation in the ER have been known as the root cause of ER storage diseases (ERSDs) (Kim and Arvan, 1998; Rutishauser and Spiess, 2002; Callea and Desmet, 2021; Padilla-Godínez et al., 2021). Aberrant protein aggregates may further impede the function of normal proteins via blocking their exit from the ER. Since the majority of ERSDs arise from gene mutations at the genetic level (Ito et al., 1999; Sørensen et al., 2006; Schaeffer et al., 2017; Hilado and Randhawa, 2018; Kuscuoglu et al., 2018; Sun et al., 2020; Wang et al., 2020), there is no curative therapy to permanently correct genetic mutation and abolish mutant protein generation. The comprehensive understanding of ER quality control network and protein aggregation process has a chance to offer valuable cues for early diagnosis and medicinal development of ERSDs.

In this work, we outline ER protein quality control system, summarize protein aggregation model and detecting tools, and exemplify the therapeutic targets of ER protein quality control machinery in two different ERSDs.

## ER protein quality control system

### Oxidative protein folding

There exists an oxidative environment involving thiol-disulfide pairs in the ER lumen, which is crucial for disulfide formation and proper protein folding (Robinson et al., 2020; Wang and Wang, 2023). The redox state of the ER also plays an important role in the modulation of calcium balance through affecting ER calcium channels and calcium-binding proteins (Ushioda et al., 2016). The majority of membrane and secretory proteins are known to contain disulfide bonds, and their folding process involves the formation of disulfide bridges between two cysteine residues. The ER has many folding enzymes and chaperones such as protein disulfide isomerase (PDI) and ER sulfhydryl oxidase 1 (Ero1) for the fidelity and efficiency of this oxidative protein folding (Wang and Wang, 2023).

The Ero1-PDI redox cycle is pivotal in ER oxidative protein folding and highly conserved across different species. The glycoprotein Ero1 introduces the formation of de novo disulfide bridge via oxidating the PDI and transferring electrons to molecular oxygen, in which hydrogen peroxide is synthesized as a byproduct. Mammalian Ero1 exists in two different types: Ero1α has a broad expression pattern, while Ero1β is present in secretory cells (Cabibbo et al., 2000; Pagani et al., 2000; Zito et al., 2010). The Ero1 oxidase activity is dynamic and under the regulation of diversely post-translational modifications and intracellular cues. When ER environment is reductive, the regulatory disulfide bonds of Ero1 are reduced by the PDI to induce its activation. Meanwhile, the phosphorylation of Ero1 Ser145 by Fam20C is recently found to increase its oxidase activity (Zhang et al., 2018). On the contrary, the regulatory bonds of the Ero1 are oxidized again to inactivate Ero1 during oxidative stress conditions. The S-nitrosylation of Cys166 in the Ero1α is capable of lowering Ero1α activity and decreasing oxidative stress (Moilanen et al., 2018; Qiao et al., 2022). The PDI is responsible for the oxidation, reduction, and isomerization of disulfide bonds in both nascent substrates and misfolded proteins. There are 21 distinct members of the PDI oxidoreductase family in mammals (Braakman and Bulleid, 2011), each of which is thought to possess a division of labor during ER oxidative protein folding. The PDIA1 has the highest expression abundance among the PDI family and is thus the dominant player in the oxidative protein folding (Li and Sun, 2021). It should be noted that the PDIs also exhibit the chaperone activity (Wang et al., 2015; Okumura et al., 2019; Yu et al., 2020). The chaperone activity of PDIs is especially useful for the inhibition of aberrant protein accumulation and the proper maturation of secretory proteins. Additionally, the largest oxidoreductase ERDj5 functions as a central player in the reduction of non-native disulfide bridges in misfolded proteins (Ushioda et al., 2008; Oka et al., 2013). Upon the interruption of aberrant disulfide bonds, faulty protein substrates can be efficiently delivered into the ERAD for degradation.

### UPR

Mammalian UPR is to sense and transmit ER stress signals to the cytosol and other organelles such as the nucleus. The UPR consists of three different signaling cascades triggered separately by protein kinase RNA-like ER kinase (PERK), activating transcription factor 6 (ATF6), and inositol-requiring protein 1α (IRE1α) (Hetz et al., 2020; Wiseman et al., 2022). All these sensors are ER membrane proteins and possess both ER-luminal and cytoplasmic domains in the meantime. Both PERK and IRE1α have kinase activity in their cytosolic region, while the cytosolic domain of ATF6 comprises a bZIP transcription factor. They are inactive in the absence of ER stresses, but rapidly exit the quiescence in the disruption of ER homeostasis. Upon stimulation of protein misfolding, the UPR sensors can be activated either by dissociating from ER chaperone BiP or by interacting directly with faulty proteins (Bertolotti et al., 2000; Zhou et al., 2006; Pincus et al., 2010; Gardner and Walter, 2011; Wiseman et al., 2022). Beyond the BiP, many molecular chaperones and proteins such as PDIs and ERp18 also participate in the activation of UPR sensors (He et al., 2012; Sepulveda et al., 2018; Oka et al., 2019; Yu et al., 2020; Wiseman et al., 2022). The three UPR pathways work together with the aim to reestablish ER proteostasis and functions.

The activation of PERK and IRE1α involves their oligomerization and autophosphorylation *in situ*, whereas the ATF6 activation is accompanied by its disulfide reduction and cleavage in the Golgi (Leitman et al., 2014; Preissler and Ron, 2019; Wiseman et al., 2022). The newly formed transcription factor ATF6(N) then travels into the nucleus where it induces the expression of ER quality control-related genes (Yamamoto et al., 2007; Adachi et al., 2008; Shoulders et al., 2013). Unlike the ATF6 pathway, the active PERK stimulates cytoplasmic eIF2α/eIF2B signaling cascade to decrease protein translation, but selectively increases the amounts of transcription factor ATF4 and C/EBP Homologous Protein (CHOP) (Lu et al., 2004; Han et al., 2013). Consequently, nascent peptides entering the ER have a significant reduction, whereas the genes controlling redox modulation and amino acid metabolism are unexpectedly increased. More complexly, three downstream signaling pathways are coupled to the activation of mammalian IRE1α (Wiseman et al., 2022). The active IRE1α primarily acts on the cytoplasmic *XBP1* transcript and abscises 26 bases by means of its endonuclease activity (Yanagitani et al., 2011; Kanda et al., 2016). The spliced *XBP1* mRNA encodes a bZIP transcription factor XBP1s to enhance the expressions of genes involved in ER protein quality control, lipid metabolism, and gluconeogenesis (Lee et al., 2003; Yamamoto et al., 2007; Shoulders et al., 2013). Meanwhile, the IRE1α could trigger the selective degradation of mRNAs and precursor miRNAs containing a consensus sequence via the regulated IRE1α-dependent decay (Hollien and Weissman, 2006; Upton et al., 2012), which reduces the protein load into the ER and boosts lysosomal flux. Thirdly, the active IRE1α may provoke the c-Jun N-terminal kinase (JNK) pathway through binding the immune sensor TRAF2 in response to severe ER stresses (Urano et al., 2000; Han et al., 2009). It is notable that both PERK and IRE1α could evoke cellular apoptotic program in the cases of acute and long-term ER stress (Wang and Kaufman, 2016; Hetz and Papa, 2018).

### ERAD

When misfolded proteins fail to be resolved by the UPR, the majority of them are cleared from the ER through the ERAD pathway (Li and Sun, 2021; Christianson and Carvalho, 2022; Thepsuwan et al., 2023). The ERAD pathway is made up of molecular chaperones, ER-membrane-resident E3 ubiquitin ligases and adaptor proteins, and cytoplasmic adaptors and proteasome. The ERAD is conserved across distinct mammalian species (Christianson and Carvalho, 2022). The recognition of ERAD substrates is implemented by ER chaperones and lectins such as BiP, glucose-regulated protein 170 (GRP170), Os9, and xanthosine 50-triphosphate-B (Plemper et al., 1997; Brodsky et al., 1999; Olivari et al., 2005; Christianson et al., 2008; Hosokawa et al., 2008; Ushioda et al., 2008; Shenkman et al., 2013). Molecular chaperones discriminate faulty protein from native protein pools by recognizing the exposed hydrophobic domain of non-glycosylated clients or by catching the trimmed glycans of glycoproteins. The PDIs are involved in the recruitment and transportation of faulty proteins with disulfide bonds (Ushioda et al., 2008; Grubb et al., 2012; Oka et al., 2013; He et al., 2015).

The retrotranslocation channels in the ER membrane transport client substrates into the cytosol for proteasome degradation. A multi-protein complex comprising the suppressor/enhancer of lin-12-like (Sel1L) and hydroxymethylglutaryl reductase degradation protein 1 (Hrd1) is the best-studied translocation channel in mammalian cells (Needham et al., 2019; Lemberg and Strisovsky, 2021; Christianson and Carvalho, 2022). The Sel1L, a type-I transmembrane protein, not only transfers client substrates to the Hrd1 but also supports the stability and activity of the Hrd1 (Olzmann et al., 2013; Sun et al., 2014; Jeong et al., 2016). Hrd1 is an ER-membrane-resident E3 ubiquitin ligase having eight transmembrane segments and can self-associate into dimeric or higher oligomeric molecules (Hwang et al., 2017; Schoebel et al., 2017; Vasic et al., 2020). A multitude of adaptor proteins such as the degradation in endoplasmic reticulum (Derlin) and HERP is involved in the organization of the Hrd1 complex and the retrotranslocation of target clients (Horn et al., 2009; Neal et al., 2018; Schmidt et al., 2020; Lemberg and Strisovsky, 2021). It has been shown that yeast Hrd1 may assemble into two-halves of a channel with the Der1, the yeast ortholog of mammalian Derlin-1 (Wu et al., 2020). Derlins are ER-membrane-resident multifunctional proteins with six transmembrane spans, while their N- and C-terminal regions face the cytosol. Notably, human Derlin-1 can form a tetrameric translocation tunnel whose cavity allows the pass of specific proteins (Rao et al., 2021), supporting the complexity of retrotranslocation channels. When client substrates access the cytosolic cavity of the Hrd1, they are ubiquitinated by the cytosolic RING finger domain of the Hrd1, thus facilitating their engagement of valosin-containing protein (VCP) for the extraction. Beyond the Hrd1, more than twenty E3 ubiquitin ligases, such as March6, TRC8, and RNF170, are implicated in the ubiquitination of ERAD substrates (Mehrtash and Hochstrasser, 2019; Lemberg and Strisovsky, 2021), which cooperate with the Hrd1 or function in parallel. The ubiquitination events driven by E3 ubiquitin ligase may get assistance from cytoplasmic E2 ubiquitin-conjugating proteins Ube2j and Ube2g2 (Christianson and Ye, 2014). Once the ubiquitinated substrates have an association with the cytosolic VCP-Ufd1-Npl4 complex, they are pulled out from the ER membrane, in which the driving force arises from ATP hydrolysis of the AAA + ATPase VCP (Pye et al., 2007; Meyer et al., 2012; Christianson and Carvalho, 2022). The VCP, known as p97, is a mechanoenzyme with two ATPase domains and participates in the retrotranslocation of nearly all ERAD substrates. The recruitment of the VCP-Ufd1-Npl4 complex to the retrotranslocation sites on the ER membrane is triggered by many ERAD factors, such as Derlin1, UbxD8, and RHBDL4 (Mueller et al., 2008; Klemm et al., 2011; Sato et al., 2019; Lemberg and Strisovsky, 2021). It should be noted that the two ATPase domains of the VCP form a hexameric ATPase ring with a central pore (Cooney et al., 2019; Twomey et al., 2019). Thus, the client substrate needs to be unfolded, at least partially, all the way when passing the VCP central pore. The disulfide bonds in disulfide-containing substrates are also disrupted to make them in the linear state (Ushioda et al., 2008; Grubb et al., 2012). The last step of ERAD pathway is transferring the retrotranslocated substrates to the cytosolic 26S proteasome, which involves many cytoplasmic chaperones such as Ubl4A and BAG6 (Ahner et al., 2007; Wang et al., 2011; Xu et al., 2012). The Ubl4A, BAG6, and Trc35 form a holdase complex to maintain the solubilization of the retrotranslocated proteins in the cytosol. The interaction between this holdase complex and the proteasome facilitates the efficient and rapid delivery of client substrates to the proteasome.

Apart from the misfolded proteins, ERAD has the capacity to manage properly folded proteins and thus governs their expression abundance (Adle et al., 2009; Bhattacharya and Qi, 2019). This kind of constitutive ERAD is independent of the UPR stimulation and functions as a protein quantity control system to maintain ER and cellular homeostasis. The endogenous substrates of constitutive ERAD include but not limited to the IRE1α, CREBH, pre-BCR, CPT2, Pgc1β, Rmnd1, Nrf2, and CDKN1B (Wu et al., 2014; Fujita et al., 2015; Sun et al., 2015; Ji et al., 2016; Xu et al., 2016; Wei et al., 2018; Wei et al., 2018). Notably, ERAD deficiency has been associated with physiological dysregulation and disease occurrence (Sun et al., 2016; Bhattacharya et al., 2018; Yang et al., 2018). The specific deletion of the Sel1L in mouse liver leads to the accumulation of transcriptional factor CREBH in hepatocellular ER, the impairment of energy metabolism, and the growth retardation (Bhattacharya et al., 2018). Likewise, the accumulation and hyperactivation of IRE1α in the Sel1L-knockout mice results in the development of colitis (Sun et al., 2015; Sun et al., 2016).

### ER-phagy

ER-phagy is an orchestrated system to remove ERAD-resistant misfolded proteins and insoluble protein aggregates from the ER via the lysosomal degradation pathway (Iavarone et al., 2022; Chino and Mizushima, 2023). Beyond the elimination of misfolded proteins, ER-phagy also plays important roles in the control of ER level and the degradation of the nuclear membrane (Chino and Mizushima, 2023). ER-phagy receptors mediate the selective recognition of ER subdomains by the cytoplasmic degradation machinery. Thus far, eleven ER-phagy receptors have been discovered in mammals: eight receptors (FAM134B, FAM134A, FAM134C, RTN3L, CCPG1, SEC62, TEX264, and ATL3) are resident in the ER membrane, and the other three receptors (CALCOCO1, C53, and p62) exist in soluble forms (Smith et al., 2018; Chen et al., 2019; Chino et al., 2019; Ji et al., 2019; Liang et al., 2020; Nthiga et al., 2020; Stefely et al., 2020; Stephani et al., 2020; Reggio et al., 2021; Stephani et al., 2021). Despite lacking transmembrane regions, soluble ER-phagy receptors may be recruited to ER membrane via the interaction with ER-membrane-resident proteins (Nthiga et al., 2020; Stefely et al., 2020; Stephani et al., 2020; Stephani et al., 2021). All of ER-phagy receptors possess at least an LC3-interacting region or GABARAP-interaction motif to bind the Atg8 family protein LC3 or GABARAP in autophagosomal membranes, respectively. Most ER-phagy receptors contain a long intrinsically disordered region to facilitate the curvature and scission of ER membranes (Busch et al., 2015; Snead et al., 2017). Growing evidence indicate that the ER-phagy receptors show preference in mediating the degradation of different ER regions and protein substrates (Grumati et al., 2017; Chen et al., 2019; Nthiga et al., 2020). For example, RTN3, ATL3, and CALCOCO1 mainly participate in the fragmentation of ER tubules, while FAM134B, TEX264, CCPG1, and SEC62 are involved in the degradation of ER sheets (Grumati et al., 2017; Smith et al., 2018; Chen et al., 2019; Nthiga et al., 2020). It is noteworthy that ER-phagy receptors exhibit diverse tissue expression patterns in mammals (Smith et al., 2018; Chino et al., 2019; Kohno et al., 2019; Keles et al., 2020).

There are three distinct forms of ER-phagy in mammalian cells: macro-ER-phagy, micro-ER-phagy, and the vesicular transport pathway (Molinari, 2021; Chino and Mizushima, 2023). Macro-ER-phagy harnesses the autophagosome to engulf and deliver ER fragments to the lysosomes, whereas the other two types of ER-phagy involve the direct fusion of ER fragments with the endosomes and/or lysosomes. During macro-ER-phagy, ER-phagy receptors mediate the recruitment of double-membrane autophagosomes to the ER subdomains containing misfolded protein aggregates (Rogov et al., 2014; Grumati et al., 2018). A set of ATG factors (e.g., ATG5, ATG7, and ULK1/2) participating in autophagosome biogenesis and ATG8 lipidation are necessary for this process. Once the autophagosomal cargos integrate into the lysosomes, protein aggregates, ER membrane, and the inner autophagosomal membrane are scheduled for degradation. The majority of ERAD-resistant protein aggregates such as vasopressin and *Akita* proinsulin mutants are disposed from the ER by this macro-ER-phagy (Cunningham et al., 2019; Forrester et al., 2019). Unlike the macro-ER-phagy, micro-ER-phagy needs the endosomal sorting complex required for transport (ESCRT) machinery rather than the autophagosome. Albeit the core autophagy factors are not involved in the micro-ER-phagy, some components of the LC3 lipidation system such as ATG4B and ATG16L1 have been observed in mammalian micro-ER-phagy during recovery from ER stresses (Schuck et al., 2014; Loi et al., 2019; Molinari, 2021). For instance, p62-dependent micro-ER-phagy is shown to handle the procollagen mutant bearing a Gly610-to-Cys (G610C) substitution in mouse cells (Omari et al., 2018). Similar to the micro-ER-phagy, the vesicular transport pathway has nothing to do with the autophagy initiation factors, but depends on the LC3-conjugation machinery (Fregno et al., 2018; Chino and Mizushima, 2020). The single-membrane vesicles detaching from the ER fuses directly with the lysosomes, which is implemented by the interaction between ER-membrane-resident syntaxin 17 and lysosomal membrane protein VAMP8 (Itakura et al., 2012; Fregno and Molinari, 2019). The Z variant of alpha1-antitrypsin (Z-AAT) with a Glu342-to-Lys (E342K) mutation is removed from the ER by this LC3-dependent transport pathway (Fregno et al., 2018; Fregno and Molinari, 2019).

### Crosstalk

The UPR, ERAD, and ER-phagy are believed to work together for the high fidelity of protein synthesis and folding in the ER. When misfolded proteins appear in the ER, UPR sensors are activated with the attempt to enhance ER protein folding, translocation, secretion and degradation. To this end, the three branches of mammalian UPR have to activate ERAD and ER-phagy at certain circumstances (Hwang and Qi, 2018; Song et al., 2018; Sun and Brodsky, 2019; Molinari, 2021). The IRE1α/XBP1s pathway has been shown to boost the transcriptional expressions of core ERAD components and autophagy gene Beclin-1 (Travers et al., 2000; Margariti et al., 2013; Song et al., 2018). Moreover, the IRE1α/XBP1s signaling can stimulate the expression of ER-phagy receptor CCPG1 via regulating transcription factor MIST1 (Smith et al., 2018; Molinari, 2021). Likewise, the PERK/eIF2α/ATF4 pathway has been found to increase the expressions of ER-phagy receptors and autophagosome-related ATG5 and ATG12 genes at the transcriptional level (B'Chir et al., 2013; Adamson et al., 2016; Song et al., 2018; Zielke et al., 2020). As such, both ERAD and ER-phagy activation following UPR stimulation assist in the maintenance of proper ER functions and contents by disposing of aberrant proteins and ER subdomains. The UPR signaling is also under the control of ERAD and ER-phagy. Both IRE1α and ATF6 have been identified as the substrates of the ERAD complex (Horimoto et al., 2013; Sun et al., 2015). For example, the Sel1L-Hrd1 ERAD can regulate normal IRE1α abundance at the protein level with the help of ER luminal chaperone BiP and Os9 (Sun et al., 2015), thus limiting the hyperactivation of IRE1α/XBP1s pathway during physiological circumstance. On the contrary, the aberrant accumulation and hyperactivation of IRE1α molecule following ERAD deficiency contribute to the development of experimental colitis and inflammation dysbiosis (Sun et al., 2015). Beyond the ERAD, ER-phagy has been shown to participate in the degradation of IRE1α oligomers when ER stress occurs (Tschurtschenthaler et al., 2017).

The ERAD has ability to cope with most misfolded proteins rather than large oligomers and aggregates. The incapacity of faulty proteins to enter the ERAD pathway may be due to their large size, the absence of ERAD-responsive signal, or their aggregation state (Fregno and Molinari, 2019). On the other side, the ER-phagy is known to handle protein aggregates as well as particular ER subregions. It should be noted that some aberrant proteins such as *Akita* proinsulin and NPC1 I1061T mutant are the targets of both ERAD and ER-phagy (Schultz et al., 2018; Cunningham et al., 2019; Molinari, 2021). In this case, their soluble monomers are delivered into the ERAD for clearance, while their large aggregates are managed by the ER-phagy pathway. Hence, the degradation selection for misfolded proteins closely depends on protein size, mutation severity, aggregation propensity, the kinetics and thermodynamics of folding, and the site of folding lesion.

## ER protein aggregation and phase transition

Although the models and mechanisms of misfolded protein aggregation within the ER remain elusive, the kinetics and research tools of protein aggregation in other cellular compartments may be applicable to the formation of ER protein aggregates. Protein aggregation in the cytoplasm is a phase transition process from soluble monomers to solid insoluble aggregates (Boeynaems et al., 2018; Dhouafli et al., 2018; Soto and Pritzkow, 2018; Wang and Roberts, 2018; Verdile et al., 2019; Zbinden et al., 2020). In the classic seeding-nucleation model, there are two sequential phases, one nucleation phase, and the other elongation stage (Dhouafli et al., 2018; Soto and Pritzkow, 2018; Wang and Roberts, 2018). During the nucleation phase, soluble protein monomers firstly assemble into non-native oligomers, which is driven by the non-covalent intermolecular interactions between hydrophobic domains or by the disulfide-mediated covalent linkage (Wang and Roberts, 2018; Franzmann and Alberti, 2019). The protein nucleation rate is closely associated with the contents of hydrophobic patches or aromatic amino acids in the monomer. The size of protein oligomers is heterogeneous from dimers to decamers, depending partially on specific protein species. Due to its reversibility and thermodynamical instability, the nucleation phase serves as the rate-limiting step in protein aggregation. Thus, the addition of oligomeric seeds is sufficient to shorten nucleation phase and accelerate protein aggregation process (Soto and Pritzkow, 2018; Wang and Roberts, 2018; Zbinden et al., 2020). In the elongation stage, oligomeric seeds can grow into large polymers quickly by recruiting misfolded and even normal proteins. Growing oligomers make use of the sticky pockets to engage other protein molecules, induce their conformational fluctuation, and then render them into the preexisting oligomeric scaffold. The growth of small protein oligomers is in an exponential manner via the monomer addition and/or oligomer-oligomer fusion (Philo and Arakawa, 2009; Wang and Roberts, 2018). As the size of protein polymers continuously increases, they could form into insoluble aggregates (Feng et al., 2019), which may involve the liquid-to-solid phase transition. The morphology of protein precipitates is manifold and determined by the degree of intermolecular arrangement (Barz and Urbanc, 2014; Mitrea and Kriwacki, 2016; Mudogo et al., 2020).

The formation of protein aggregates is concentration dependent. In theory, a high protein concentration can decrease intermolecular interaction distance and thus accelerate protein aggregation. Once the concentration of accumulated proteins reaches over the nucleation threshold, protein aggregation should take place instantaneously. Indeed, the high concentration of κ chain of human immunoglobulin G2 (IgG2) in a neutral pH solution is found to self-assemble into protein crystals *in vitro* (Hasegawa et al., 2011; Hasegawa et al., 2014). Overexpressing human IgG2/κ protein in CHO cells leads to the accumulation of IgG2/κ molecules and the formation of rod-shaped protein crystals in the ER (Hasegawa et al., 2011). The overexpression of human neuraminidase-1 cleaving the N-terminal sialic acids of glycoconjugates allows spontaneous formation of cuboid protein crystals in the ER of human embryonic kidney 293 cells (Hasegawa, 2019). Unlike faulty protein aggregates, these intra-ER protein crystals are made of properly folded proteins whose accumulation results from the inability of the ER to export the excess molecules. Recently, the overexpressed Z-AAT molecules are shown to form a solid protein matrix in the ER of CHO cells and retard the motility and functions of ER proteins (Chambers et al., 2022). These observations suggest that protein aggregation in the ER may be a liquid-solid phase separation event.

### Detecting methods for protein aggregation and phase separation

There exist limitable approaches to characterize protein aggregates (Hamrang et al., 2013; Mitrea and Kriwacki, 2016; Wang and Roberts, 2018; Sinnige et al., 2020), most of which are used to measure protein aggregation *in vitro*. Since it is difficult to forecast when and where the aggregation of faulty proteins happens in multi-cellular organisms, the *in vivo* assessment of protein aggregation is extremely challenging. Thus, the spontaneous aggregation of purified recombinant proteins *in vitro* provides a chance to assess the kinetic process of protein aggregation.

Size exclusion chromatography (SEC), dynamic light scattering (DLS), analytical ultracentrifugation (AUC), and atomic force microscopy (AFM) have been employed to capture the early aggregation events of soluble protein oligomers (Mahler et al., 2009; Hamrang et al., 2013; Wang and Roberts, 2018). Each detection tool has its advantages and limitations. The SEC is sensitive and reproducible, but only detects small oligomers less than 50 nm. Besides, the necessary dilution of detection samples in the SEC application may trigger the dissociation of reversible protein aggregates. The DLS is a non-destructive technique with high sensitivity to large protein aggregates, but is unsuitable for polydisperse samples. The AUC could be taken to identify the content of protein aggregates upon its high resolution, but its wide application is limited by the low throughput and intricate data analysis. Importantly, the combinational application of SEC and DLS offers an effective strategy to monitor the size alteration of protein oligomers, while the SEC together with AUC would be very useful in characterizing the components of small protein aggregates. Regarding insoluble protein aggregates, the visual inspection can be used to identify visible protein particles via the evaluation of opalescence and turbidity, whereas light obscuration, flow imaging analysis, and microscopic technology have been harnessed to detect subvisible protein aggregates (Mahler et al., 2009; den Engelsman et al., 2011; Mitrea and Kriwacki, 2016; Wang and Roberts, 2018). Light obscuration measurement is a rapid quantitative method, but likely generates false-positive outcomes and not works for translucent particles. The flow imaging analysis is highly sensitive in analyzing the morphology of subvisible protein aggregates less than 400 microns, but its mass data volume restrains its broad application. Unlike the low resolution of optical microscopy measurement, fluorescence microscopic analysis has ability to characterize the morphology and size of protein aggregates, and its high sensitivity enables it to monitor early-stage protein aggregation. As extrinsic fluorescence dyes in fluorescence microscopic analysis may disturb protein aggregation at some extent, other detecting tools should be synergistically applied with the fluorescence microscopic analysis for the identification of protein aggregates.

Since the *in vitro* characterization of protein aggregation may mistakenly recapitulate protein aggregation process *in vivo*, the overexpression of fluorescently labeled proteins in cell line has been utilized to characterize the dynamic features of protein aggregation in living cells (Frottin et al., 2019; Hasegawa, 2019; Sinnige et al., 2020; Zbinden et al., 2020). Single-molecule fluorescence correlation spectroscopy (smFSC), single-molecule förster resonance energy transfer (smFRET), and electron paramagnetic resonance (EPR) spectroscopy are found to be effective in profiling the formation of protein aggregates in the cellular models. Notably, the inducible expression of fluorescently tagged proteins in the cellular model has been described to visualize the early events of protein aggregation (Miyazaki et al., 2016). Moreover, fluorescence recovery after photobleaching (FRAP) technique enables the observation of the kinetic addition of fluorescent protein molecules into protein aggregates in the living cells (Wang et al., 2013; Mitrea and Kriwacki, 2016). Both electron microscopy and immunohistochemistry technology have been taken to determine the relative amount of protein aggregates *in vivo* (Sung et al., 2015; Noda et al., 2020; Zbinden et al., 2020). Electron microscopy such as transmission electron microscopy and 4-dimensional ultrafast electron microscopy utilizes the greyscale contrast of particles to determine protein aggregates, whereas the immunohistochemistry analysis needs specific protein antibodies to measure the aggregated proteins. Given the limitation of a single detection tool, one detection technology is unable to cover all features of protein aggregation. Thus, the combination of multiple detecting tools should be adopted to gain insight into the protein aggregation.

## Therapeutic applications in the ER storage diseases

Albeit the ER devotes numerous efforts to safeguard protein fidelity, protein misfolding and accumulation may appear in the ER owing to gene mutations, oxidative stress, or nutrient deprivation. The aggregation of misfolded proteins and the dysfunction of the ER play crucial roles in the development of ERSDs. Thus far, more than 11 types of ERSDs have been identified in humans (Kim et al., 1996; Ito et al., 1999; Brennan et al., 2002; Marini et al., 2007; Schaeffer et al., 2017; Hilado and Randhawa, 2018; Wang et al., 2020; Li and Sun, 2021). The retention of defective protein aggregates in the ER is a typical characteristic of these ERSDs. Notably, the enhancement of ERAD or ER-phagy activity has been reported to boost the clearance of mutant protein aggregates from the ER in preclinical animal models of human ERSDs (Hidvegi et al., 2010; Kaushal et al., 2010; Pastore et al., 2013; Xu et al., 2018; Miyata et al., 2020). This selective degradation of pathological protein aggregates suggests the therapeutic potential of increasing ER protein clearance activity in the ERSDs. Since some ERSDs have been discussed elsewhere (Kuscuoglu et al., 2018; Callea et al., 2021; Li and Sun, 2021), here we pay more attention on two representative forms of ERADs.

### Proopiomelanocortin (POMC) mutation-driven early-onset obesity

POMC mutation-driven obesity is a recessive genetic disease characterized by hyperphagia and excessive body weight (Creemers et al., 2008; Hilado and Randhawa, 2018). The POMC is a multivalent polypeptide synthesized in the ER sheets. Once entering the secretory granules, native POMC is split into around 10 bioactive peptides such as α-melanocyte-stimulating hormone (α-MSH) and β-endorphin under the action of prohormone convertases (Millington, 2006; Harno et al., 2018). POMC derivatives are essential for numerous physiological processes including but not limited to the appetite, melanocyte stimulation, immune modulation, and skin pigmentation. For example, α-MSH is the major POMC derivative in hypothalamic neurons and acts as a key player in regulating food intake and energy balance (Kim et al., 2018; Fujima et al., 2020). Upon the importance of POMC derivatives, the deficiency of functional POMC and derivatives leads to hyperphagia, obesity, hypopigmentation, and adrenal insufficiency (Creemers et al., 2008; Hilado and Randhawa, 2018).

The folding of POMC molecules is error prone due to the presence of multiple disulfide bonds, but faulty POMC in the ER can be timely removed by the ERAD pathway during normal conditions. On the contrary, pathogenic POMC-C28F mutant harboring a cysteine-to-phenylalanine mutation at position 28 escapes ERAD-mediated destruction and eventually forms large aggregates inside the ER (Creemers et al., 2008; Kim et al., 2018). The POMC-C28F mutant is unstable because of its phenylalanine side chain, so the intermolecular disulfide linkage mediates its spontaneous aggregation (Kim et al., 2018). Supporting this notion, the creation of an intragenic cysteine-to-serine or cysteine-to-alanine mutation at position 50 is found to abolish the aggregation of POMC-C28F mutant (Kim et al., 2018; Bhattacharya and Qi, 2019). As the C28F mutation resides in the N-terminal domain guiding POMC trafficking, POMC-C28F mutant can't be transported into the secretory granules and is stuck in the ER (Creemers et al., 2008). The incapacity of POMC-C28F mutant to generate functional derivates eventually results in the occurrence of hyperphagia and severe obesity (Creemers et al., 2008; Kim et al., 2018).

The disease progression of POMC mutant-driven obesity can be recapitulated in the mice with either ERAD or autophagy deficiency (He et al., 2015; Kim et al., 2018). When the Sel1L is specifically deleted in the POMC neurons, nearly all POMC molecules are detained in the ER of neural cells, leading to the shortage of POMC derivatives in Sel1L-knockout mice (Kim et al., 2018). The mice with Sel1L deletion become hyperphagic and ultimately develop age-associated obesity, hyperleptinemia, and insulin resistance (Kim et al., 2018). Likewise, the deletion of autophagy-related ATG5 or ATG7 in hypothalamic POMC neurons is found to trigger abnormal glycolipid metabolism and severe obesity in the ATG5-or ATG7-deficient mice (Coupe et al., 2012; Kaushik et al., 2012; Malhotra et al., 2015), suggesting the involvement of ER-phagy in the degradation of misfolded POMC. Recently, the macro-ER-phagy driven by RTN3 has been reported to battle with POMC-C28F mutant aggregates (He et al., 2015). At present, there is no curative option for POMC-mutant-driven obesity. The application of melanocortin-4 receptor agonist setmelanotide has been manifested to reduce food uptake and induce substantial weight loss in patients with POMC deficiency (Table 1) (Kühnen et al., 2016; Clément et al., 2020). However, the setmelanotide treatment is offered only for the mitigation of secondary symptoms of genetic obesity without removing the *POMC* gene mutation. Given the pivotal roles of ER quality control system in aberrant protein clearance, it is worth exploring whether the enhancement of ERAD or ER-phagy activity may efficiently treat genetic obesity caused by POMC mutations.

**TABLE 1:** Promising therapeutic strategies for representative ERSDs.

Disease	Treatment	Model	Mechanisms	Effects
Early-onset obesity	Setmelanotide	Patients with POMC deficiency	Increases the satiety	Weight loss ↑
			Boosts systemic metabolism	
AATD	Carbamazepine	Z-AAT transgenic mice	Increases Z-AAT degradation	Hepatic fibrosis ↓
			Enhances autophagy flux	
	Rapamycin	Z-AAT transgenic mice	Reduces Z-AAT accumulation	Hepatic fibrosis ↓
			Increases autophagic activation	
	Norursodeoxycholic acid	Z-AAT transgenic mice	Inhibits Z-AAT aggregation Promotes autophagy flux	Hepatic damage ↓ Hepatic apoptosis ↓
	TFEB ovexpression	Z-AAT trangenic mice	Enhances Z-AAT clearance Upregulates autophagy activity	Hepatic fibrosis↓
	Hrdl ovexpression	Cellular model	Increases Z-AAT degradation	Unclear

### Alpha1-antitrypsin deficiency (AATD)

The congenital AATD is caused by genetic mutations in the *SERPINA1* gene encoding the alpha1-antitrypsin (AAT) (Sveger, 1976; Karatas and Bouchecareilh, 2020; Strnad et al., 2020). The AAT is a secretory glycoprotein whose folding and modification occur in the ER of hepatic cells. Once maturation, the AAT leaves the CNX/CRT cycle and is secreted into extracellular space via the secretory pathway. AAT is the most abundant serine protease inhibitor in the bloodstream and functions to protect distant tissues from protease-mediated destruction (Esra et al., 2019; Karatas and Bouchecareilh, 2020). Nevertheless, pathological AAT mutants are unable to be recognized by the ER-Golgi intermediate compartment that delivers native AAT from ER to Golgi (Callea et al., 1992; Leon and Bouchecareilh, 2021), thereby causing their retention in the hepatocyte ER. Due to the insufficiency of functional AAT, AATD patients suffer from pulmonary emphysema and liver cirrhosis.

Among over 100 AAT mutations, the Z-AAT is the most severe mutant affecting ∼1 in 1,500 newborns worldwide (Karatas and Bouchecareilh, 2020; Leon and Bouchecareilh, 2021). The folding of Z-AAT mutant is slow even with the assistance of many ER chaperones (e.g., BiP, GRP170, and PDIs), so Z-AAT molecules yield fault conformation and are stuck in the ER. Although both ERAD and ER-phagy can deal with Z-AAT mutants (Qu et al., 1996; Teckman and Perlmutter, 2000; Teckman et al., 2001; Kruse et al., 2006; Fregno et al., 2018), the continuous generation of Z-AAT eventually overwhelms the clearance ability of ER quality control machinery. As a consequence, the Z-AAT mutant accumulates in the ER lumen and self-assembles into large aggregates by the classic loop-sheet model or by the C-terminal domain swap mechanism (Shin and Yu, 2002; Yamasaki et al., 2011; Karatas and Bouchecareilh, 2020). It should be noted that inclusion body is found in the hepatocytes of AATD individuals (Granell and Baldini, 2008; Granell et al., 2008). Inclusion bodies are a small portion of ER sheets and are filled up with insoluble Z-AAT aggregates. The inability of the inclusion body to sequestrate Z-AAT aggregates has been shown to impair normal protein trafficking in the ER and evoke cell shrinkage (Granell et al., 2008), suggesting the protective roles of inclusion body formation in ER functions. Moreover, the durative accumulation of Z-AAT aggregates triggers oxidative stress, impairs calcium balance, and provokes inflammatory reactions in the liver (Lomas et al., 1992; Miranda et al., 2010; Bouchecareilh, 2020), thereby contributing to hepatic dysfunctions and other organ damages.

Although functional AAT administration can be used to treat AATD patients with emphysema (Wewers et al., 1987; Sandhaus, 2004), this augmentation treatment is shown to not improve lung function and reduce exacerbation events in AATD patients with obstructive pulmonary disease (Chapman et al., 2015; Stolk et al., 2019). Hence, it is necessary to develop novel therapeutic approaches for the treatment of AATD patients. The augment of ERAD or ER-phagy activity may represent a promising therapeutic strategy for AATD (Table 1). Consistent with the clearance of Z-AAT aggregates by FAM134B-driven ER-phagy (Fregno et al., 2018), the specific overexpression of autophagy regulator TFEB in mouse liver is found to suppress Z-AAT aggregation and alleviate hepatic damages in the Z-AAT transgenic mice (Pastore et al., 2013). The systemic application of autophagy inducer rapamycin, carbamazepine, or norursodeoxycholic acid in the Z-AAT transgenic mice has been described to ameliorate liver fibrosis and inflammation via enhancing autophagic flux and reducing Z-AAT aggregates (Hidvegi et al., 2010; Kaushal et al., 2010; Tang et al., 2016; Wang et al., 2019). Additionally, the enhancement of ERAD activity by overexpressing the Hrd1 could increase Z-AAT degradation and decrease intracellular A-AAT accumulation in the cellular model (Wang et al., 2011).

## Perspectives

Despite recent advances, there are many open questions to be addressed in the future. The identification of mutant protein aggregates and inclusions in the ER is qualitative without the description of dynamic protein aggregation. Thus, more studies are needed to explore whether the aggregation of misfolded proteins in the ER follows the seeding-nucleation model. The cellular models have been taken to investigate ER protein aggregation by stably overexpressing protein in living cells (Hasegawa et al., 2011; Hasegawa et al., 2014; Hasegawa, 2019), but they aren’t the ideal systems to mirror the *in vivo* process of protein aggregation. The stable overexpression of specific protein in cell lines may facilitate protein aggregation behavior via inducing protein overcrowding and cellular stress, which contributes to the artificial peculiarity of cellular models. In this scenario, it will be reasonable to employ primary cell culture or zebrafish model to investigate the dynamic features of ER protein aggregation *in vivo*. Moreover, as liquid-liquid phase separation conduces to the formation of membraneless compartments in eukaryotic cells (Feng et al., 2019; Noda et al., 2020; Zbinden et al., 2020), it is interesting to assess whether the liquid-liquid phase separation participates in the aggregation of ER-resident proteins. Another valuable direction is to measure the contribution level of each ER quality control pathway to the degradation of misfolded and aggregated proteins *in vivo*, especially in animal disease models. Since the UPR, ERAD, and ER-phagy exist in all mammalian cells, the feasible path to address the above issue is utilizing the animal model with deletion of at least one ER quality control pathway. The aging has been associated with ER stress and UPR impairment (Chadwick and Lajoie, 2019; Bhattarai et al., 2020; Taylor and Hetz, 2020). Chronic ER stress and the impaired UPR activation might be important inducers in the death of aged cells during aging. The upregulation of UPR activity by overexpressing XBP1s is reported to extend the life span of *C. elegans* (Taylor and Dillin, 2013), while the XBP1s overexpression in aged mouse brain significantly improves age-related behaviors and brain dysfunctions in murine (Cabral-Miranda et al., 2020). As the linkage between aging and ERAD or ER-phage remains elusive, it is necessary to investigate how the aging changes ER quality control pathways and how the manipulation of ERAD or ER-phagy regulates the aging process. It should be noted here that the improper activation of UPR pathways conduces to the progression of some human diseases including the diabetes, viral infection, and hepatic steatosis (Chen et al., 2008; Kammoun et al., 2009; Allagnat et al., 2012; Han et al., 2015; Li et al., 2019; Bhattarai et al., 2021). For example, the hyperactivation of IRE1α and ATF6 signaling pathways has been found to induce pancreatic cell death and exacerbate insulin resistance in the animal models of diabetes (Chen et al., 2008; Allagnat et al., 2012; Han et al., 2015). In these circumstances, it is highly reasonable to suppress the activity of UPR signaling for the efficient alleviation of disease symptoms. However, more researches are needed to explore whether inhibiting the UPR is beneficial to health in animal disease models or clinical settings. With the advent of novel detection techniques, this exploration during pathological conditions holds great promise for the development of medicinal targets in ER protein quality control system.
